# Polychlorinated Biphenyls in Brackish Water Fish in the River Niger, Nigeria

**DOI:** 10.5696/2156-9614-8.17.31

**Published:** 2018-03-12

**Authors:** John P. Unyimadu, Oladele Osibanjo, Joshua O. Babayemi

**Affiliations:** 1 Nigerian Institute for Oceanography and Marine Research, Victoria Island, Lagos, Nigeria; 2 Department of Chemistry, University of Ibadan, Nigeria; 3 Department of Chemical and Food Sciences, Bells University of Technology, Ota, Nigeria

**Keywords:** polychlorinated biphenyls (PCBs), brackish water fish, pollution, River Niger

## Abstract

**Background.:**

Anthropogenic polychlorinated biphenyls (PCBs) in aquatic environments poses human and ecological health risks in Nigeria.

**Objectives.:**

This study determined the concentrations of PCBs in brackish water fish in the River Niger to assess the contamination status of fish consumed by the local population.

**Methods.:**

The sampled fish species included Drepane africana, Mochokus niloticus, Chrysichthys nigrodigitatus, Pristipoma jubelini, Vomer septapinis, Pseudotolithus senegalensis, Mugil cephalus, Pseudotolithus elongatus, Sphyraena piscatorum and Lutjanus goreensis, purchased from landing sites. Six fish from each species were sampled, for a total of 60 samples. Twenty-seven (27) PCB congeners, #8, #18 #28, #44, #52, #60, #77, #81, #101, #105, #114, #118, #123, #126, #128, #138, #153, #156, #157, #167, #169, #170, #180, #185, #189, #195, and #206 were screened in the fish samples using standard methods. The PCBs were identified and quantified using gas chromatography (GC) (Hewlett Packard GC 5890 series 11 with electron capture detector). Confirmation was performed using Shimadzu GCMS QP2010.

**Results.:**

The sum of the National Oceanic and Atmospheric Administration Agency (ΣNOAA) PCBs occurred at the highest concentration of 1830.0±484.0 μg/kg detected in Vomer septapinis, and the lowest in Pseudotolithus senegalensis, with a mean concentration of 795±169.3 μg/kg. The concentration of dioxin-like (DL) PCBs was highest in Pristipoma jubelini (992.0±88.6 μg/kg) and lowest (285.6±81.5 μg/kg) in Drepane africana. The highest mean concentration (418.±177.6 μg/kg) of International Council for the Exploration of the Seas-7 (ICES-7) PCBs was observed in Vomer septapinis. The heavier ICES-7 congeners PCB-138, PCB-153, and PCB-180 occurred at higher concentrations compared to the lighter molecular weight ICES-7: PCB-28, PCB-52, and PCB-101. The European Union (EU) marker PCB limit of 335 μg/kg was exceeded in all the brackish water fishes with the exception of Mochokus niloticus, Pristipoma jubelini and Pseudotolithus senegalensis.

**Discussion.:**

The total level of PCBs in the brackish fish samples was relatively high at >1000 μg/kg (above the World Health Organization (WHO) and Food and Agriculture Organization of the United Nations (FAO) guideline of 1000 μg/kg fresh weight). The EU guideline value for fish (220 μg/kg fresh weight) was exceeded in about 80% of the brackish fish samples studied.

**Conclusions.:**

Consumption of fish from the River Niger may expose humans to polychlorinated biphenyls. In addition, since contamination of the fish samples is an indication of river contamination, river water quality is of great concern and there is a need for additional PCB data on water quality to be distributed to the community, followed by mitigation measures.

**Competing interests.:**

The authors declare no competing financial interests.

## Introduction

Improper waste management and pollution are environmental problems confronting developing countries such as Nigeria. The effects of pollution are seen across the various components of the environment.^1^,^2^ Contamination of aquatic products and food items with organic pollutants, especially organochlorine pesticides, have been reported.^3^ Other organic pollutants which require assessment are polychlorinated biphenyls (PCBs) due to their historically intensive use in the region.

Manufactured in different countries under various trade names (e.g., Aroclor, Clophen, Phenoclo), PCBs were introduced in 1929. These groups of organic pollutants are chemically stable and heat resistant, and were used worldwide as transformer and capacitor oils, hydraulic and heat exchange fluids, and lubricating and cutting oils.^4^ Theoretically, a total of 209 possible chlorinated biphenyl congeners exist. The congeners consist of ten homologues (1–10): mono, di, tri, tetra, penta, hexa, hepta, octa, nona, and deca. The different homologues have 1–46 PCB isomers with similar molecular weight. The sequence in isomeric forms are penta-46, tetra, hexa-42 each, tri, hepta-24 each, di, octa-12 each, mono, nona-3 each and deca-1, for a total of 209 isomers.^5^ Out of the 209 possible PCB congeners, only about 130 are likely to occur in commercial products. However, these chemicals are being phased out of use in products because of their health effects and persistence in the environment.

The persistence of PCBs may be understood by the mode and mechanism of their degradation. Biodegradation of PCB congeners occurs by dechlorination by aerobic and anaerobic bacteria.^5^ Generally, these are slow processes and hence contribute to the persistence of PCBs. The low congeners of mono, di, and tri PCBs generally biodegrade aerobically, while the heavier congeners (tetra, penta, hexa PCBs) generally biodegrade anaerobically. Biodegradation also depends on temperature and PCB concentration.

PCBs have been classified as substances for which there is evidence of endocrine disruption.^6^,^7^ There is, therefore, the need to monitor the presence and levels of these chemicals in the environment, particularly in aquatic products through which humans may be exposed.

High levels of PCBs in the water of the River Niger have recently been reported, along with other persistent organic pollutants (POPs) such as organochlorine pesticides.^8^,^9^ There is a need to determine if PCBs are bioaccumulated in the fish species of the River Niger. This study, therefore, aims to determine PCB levels in the brackish water fish of the River Niger.

## Methods

Appropriate locations for the collection of fish samples were selected, and sample types and species and sample numbers for each location were identified. Samples were collected during the fall when reproduction was deemed to be complete and a full dry season of exposure to potential toxins had occurred. The target species of fish in this project were representative and economically important species for the River Niger.

The sampled fish were identified as either demersal, benthic pelagic or pelagic. Sampled fish species included Drepane africana, Mochokus niloticus, Chrysichthys nigrodigitatus, Pristipoma jubelini, Vomer septapinis, Pseudotolithus senegalensis, Mugil cephalus, Pseudotolithus elongatus, Sphyraena piscatorum and Lutjanus goreensis.

Fish samples were purchased from landing sites. The method of fishing in those areas included netting, trotlines, angling, and traps. The fish were thoroughly washed with distilled water, sorted and identified. They were placed in food grade coolers, covered with ice blocks and as quickly as possible shipped to the laboratory for analysis where they were deep frozen immediately on arrival in the laboratory. In the laboratory, basic biogenic data were generated, including total length (measured to the nearest tenth of a centimeter) using a metric measuring board, and weighed to the nearest gram using a metric weighing scale. Observations were made on other conditions of the fish, e.g. tumors, lesions, collection problems, weather conditions, etc. The knife used for filleting was washed with soapy water and rinsed with analytical grade acetone (both sides of the knife blade were decontaminated), air-dried and wrapped in aluminum foil and kept inside zip closure plastic bags. The knife was decontaminated between samples.

**Abbreviations**
*ADI*: Acceptable daily intake
*BDL*: Below detection limit
*DL*: Dioxin-like
FAO: Food and Agriculture Organization of the United Nations
*GC*: Gas chromatography
*ICES-7*: International Council for the Exploration of the Seas-7 Indicator PCBs
*NOAA*: National Oceanic and Atmospheric Administration Agency
TEF: Toxicity equivalent factor
*TEQ*: Toxic equivalent
*WHO*: World Health Organization

Latex gloves were worn during the handling of the fish for fillet processing. Fish were rinsed with distilled water before scaling and filleting. The samples were carefully scaled on a fillet board lined with heavy duty aluminum foil. Both sides of the fish were filleted, including the belly portion. After filleting the fish, the fillet was wrapped in restaurant grade aluminum foil with the dull side in contact with the skin and placed in a zip closure plastic bag with the sample label dropped inside. The skin-off fillet was collected for analysis in the present study. After filleting, a homogenous tissue sample was created using a commercial-grade food blender washed with phosphate-free soap and water and rinsed with hexane between samples to avoid cross contamination. Each sample was ground three times, with the tissue mixed between grinding to ensure a homogenous sample. Two packets of tissue were prepared and wrapped in aluminum foil, numbered and frozen. One packet was used for PCBs analysis and the other for pesticide analysis.

### Reagents

Analytical grade hexane, acetone, dichloromethane, petroleum ether, and acetonitrile were purchased from Merck (Germany). They were distilled over a 0.5-m packed column (reflux ratio approximately 1:25).^10^ Solvent purity was tested by gas chromatographic analyses. Anhydrous granulated sodium sulfate and silica gel 100–200 mesh (Merck, Germany) were cleaned with pure n-hexane by distillation. The external and internal standard were purchased from Restek (USA) and constituted of 1000 μg/ml of the following PCB congeners: #8, #18, #28, #44, #52, #60, #77, #81, #101, #105, #114, #118, #123, #126, #128, #138, #153, #156, #157, #167, #169, #170, #180, #185, #189, #195, and #206.

### Sample Preparation

Samples were extracted in batches of 6–8 samples including all the necessary quality assurance/quality control (QA/QC) measures specified in the method used for this study. All of the solvents were pesticide quality grade. Fish tissue previously homogenized using a commercial grade food blender (washed with phosphate-free soap and water and rinsed with hexane between samples to avoid cross-contamination) was thawed and thoroughly mixed. Then 5 g of tissue was weighed into a contaminant-free 150 mL Pyrex Berzelius beaker and mixed with 20 g of anhydrous sodium sulfate (sodium sulfate granular, Supelco 2-0296) which had been previously dried by heating to 140°C overnight. The mixture was stirred frequently, until it was dry and free-flowing, containing no large lumps. The beakers were then numbered with appropriate sample numbers and weighed. Decafluorobiphenyl (15 μg/L) purchased from AccuStandard (USA) was added as the surrogate internal standard.

### Extraction

The extraction columns (330 mm × 23 mm, each fitted with removable Teflon stopcock) were prepared by inserting a small glass wool (Corning Pyrex) plug into the bottom of each chromatographic column and then the column was rinsed twice with 15 mL of petroleum ether. Air was removed from the glass wool by lightly tapping it with a clean glass stirring rod. A Zymark concentration tube, 200 mL with 1 mL endpoint, was placed under each column and the appropriate labels were transferred to these tubes. The sample mixture was then poured into the column, after which 50 mL of acetone/petroleum ether was added to the sample beaker, stirred and transferred to the column. The solvent was allowed to pass through the column, but as it began to elute into the concentration tube, the stopcock was closed. At this point, the column was lightly stirred with a glass rod to remove trapped air. Elution was then continued at the rate of 1–2 mL per minute until the solvent level reached the beginning of the sample mixture. Another 50 mL of acetone/petroleum ether was added and elution was continued at the same rate. The columns were allowed to drain completely after the second 50 mL of solvent was added. The stopcocks were rinsed with acetone/petroleum ether to wash any residue lipids or analytes into the concentration tube.

### Extract Concentration

The eluent was concentrated by placing the concentrator tubes in a Kuderna-Danish TurboVap concentrator. The concentrator water bath was kept at 40°C and the argon sweep gas (purified grade) pressure was set at 10–12 psi with the TurboVap control knobs. The sample was concentrated to 1 mL and solvent exchanged to isooctane. The extract was transferred to a 2-ml graduated vial with isooctane.

### Lipid Determination

The method of lipid analysis defined by Sloan *et al.* was used.^11^ Prior to clean up by column chromatography, a volume of sample extract equivalent to 1 g of tissue was pipetted into a pre-weighed (after acetone rinsing and drying) aluminum drying pan. Pre-weighing was performed to the nearest 0.1 mg. The extract was allowed to evaporate under static conditions in a fume hood for 2 hours. The pan was weighed again to the nearest 0.1 mg and the percent extractable lipid was computed as 100 × (1-weight of residual lipid).

### Sample Cleanup

A 600-mm × 19 mm cleanup column was prepared by blocking the hole with glass wool and adding 3 g of activated silica gel (60 to 100 mesh, calcined at 650°C for 24 hours in a muffle furnace, and then stored at 130°C until use). Before use, the silica gel was deactivated with 1 ml distilled water. The column was topped with 1 cm of preheated sodium sulfate previously heated at 650°C for 8 hours in a furnace, and stored in a clean bottle in a desiccator. The column was rinsed by eluting with 20 ml hexane twice and discarded. The concentrated extract in iso-octane was transferred to the column and eluted with 50 ml of 20 + 80 DCM/hexane (v/v ratio). The eluent was collected in a 100 ml round bottom flask. This fraction contained PCBs.^12^ The eluates were reduced by volume with rotary evaporator to 3 ml and solvent exchanged to iso-octane and the volume further reduced to 1 ml in a stream of nitrogen.^12^

### Quality Control and Data Analysis

Certified reference standards from AccuStandards (USA) were used for the instrument calibration and quantification of PCB congeners. The PCB congeners were identified in the sample extracts by comparing the accurate retention time from the standard mixture and quantified using response factors from five level calibration curves of the standards. Appropriate QA/QC analysis was performed including analysis of procedural blanks (analyte concentrations were below method detection limit) to check for purity of reagents, potential laboratory contamination and inferences. Random duplicate samples were analyzed (standard deviation <5) to check the precision of the instrument. Five calibration curves with r2 values of 0.999 were used for quantification of PCBs. Calculated concentrations were reported as less than the limit of detection if the peak area did not exceed the specified threshold (three times the noise). Concentrations below the detection limit (BDL) were assigned zero values for the statistical analysis. The PCBs were denoted by their International Union of Pure and Applied Chemistry numbers. The certified reference material from the International Atomic Energy Agency was extracted, cleaned up and analyzed using the same procedure used for the environmental samples. All the results were expressed in wet weight bases and were not corrected for recoveries.

### Analytical Methods

Twenty seven (27) PCB congeners, #8, #18 #28, #44, #52, #60, #77, #81, #101, #105, #114, #118, #123, #126, #128, #138, #153, #156, #157, #167, #169, #170, #180, #185, #189, #195, and #206 were screened in the fish samples. The analytical standards (>98% purity) were used to prepare fortification and standard solutions. The extracted samples were subjected to gas chromatography for identification/quantification. The compounds were analyzed using a Hewlett Packard gas chromatography (GC) 5890 series 11 with electron capture detector. The 1-μL sample was split to two columns, 0.53 mm × 30 m each, containing SPB-5 and SPB-608 stationary phases, respectively, for the confirmation of the peaks to detect co-elution peaks, particularly PCB-77/110 and PCB-138/163. The instrument was operated in splitless mode, (closed for 1.5 minutes) and the oven temperature program started at 90°C (held for 2 minutes) to 130°C at 15°C/minute, then to 290°C at 4°C/minute (holding time 20 minutes). Injector and detector temperatures were 250 and 300°C, respectively. The flow rate through the column was 3 mL minute-1. Calibration curves were performed for quantification of each compound.

The model and type of GC used in the confirmation study was a Shimadzu GCMS QP2010 and capillary column type: HP1MS (30m × 0.25um × 0.25mm). The GC was checked to ensure that it was in good condition. Thereafter, it was flushed with the carrier gas. Calibration was done using reference standards; 0.063; 0.125; 0.25; 0.5; 1.0 ppm. These standards were run six times to calculate the mean, range, and standard deviation along with peak column performance, peak height and resolutions. Helium gas was used because of its inertness. The statistical data analysis of the results included t-tests and correlations using the Statistical Package for the Social Sciences (SPSS) software package.

## Results

The biometric data are shown in Table 1 and include biological name, sample location, fish total weight, total length, % fat and moisture content. A maximum mean length of 60 cm was observed for Sphyraena piscatorum. The minimum length observed for Drepane africana was 14.0±1.0 cm. Maximum and minimum fish weights of 3200 and 109±11.3 g were observed for Lutjanus goreensis and Mochokus niloticus, respectively. The highest fat content was observed for Sphyraena piscatorum (6.60%), while the lowest fat content was observed for Pristipoma jubelini (1.18%). The moisture content was high in the samples with a maximum of 75.11% observed for Pristipoma jubelini.

**Table 1:** Biometric Data of Fish Samples

**Species**	**Fish Weight (g)**	**Fish Length (cm)**	**% Fat**	**% Moisture Content**
Drepane africana	300±50.0 (250–350)	14.0±1.0 (13.0–15.0)	1.22±0.94 (0.28–2.15)	73.8±1.45 (72.4–75.3)
Mochokus nilotiwcus	109±11.3 (95.0–130)	19.5±0.88 (18.0–21.0)	4.04±1.08 (2.49–6.16)	72.9±0.92 (71.3–74.3)
Chrysichthys nigrodigitatus	1470±111 (1300–1600)	48.0±6.00 (39.0–53.0)	6.12±0.78 (5.12–7.30)	72.2±0.57 (71.3–73.0)
Pristipoma jubelini	250	15.50	1.18	75.11
Vomer septapinis	150±50.0 (100–250)	15.5±2.00 (12.0–19.0)	3.42±2.16 (1.02–6.73)	73.9±2.01 (71.6–76.3)
Pseudotolithus senegalensis	150±50.0 (100–200)	17.6±0.75 (17.5–18.0)	4.62±0.22 (4.40–4.83)	72.7±0.28 (72.4–73.0)
Mugil cephalus	148±23.8 (100–180)	16.2±6.70 (15.0–17.0)	3.24±1.38 (1.13–6.01)	73.6±1.68 (71.4–75.2)
Pseudotolithus elongatus	262±55.2 (200–400)	28.0±3.20 (21.0–34.0)	2.32±1.46 (0.30–4.30)	74.3±0.79 (73.0–75.5)
Sphyraena piscatorum	350	60.0	6.60	71.43
Lutjanus goreensis	3200	56.00	2.25	69.13

The brackish water fishes identified and analyzed in this project were classified into 8 families: Drepaneidae Bagridae, Haemulidae, Carangidae, Sciaenidae, Mugilidae, Sphyraenidae and Lutjanidae. All the samples were demersal, ie living close to the bottom of the river. The maximum attainable length of the fishes ranged between 45 cm to 120 cm; the family Sciaenidae, Mugilidae and Sphyraenidae (e.g. Pseudotolithus senegalensis, Mugil cephalus, and Sphyraena piscatorum) can grow to a maximum length between 115 to 205 cm. The shorter species are Drepaneidae and Carangidae, at 38 to 40 cm. All the samples were native to the River Niger and belonged to different trophic levels.

**Figure 1:**
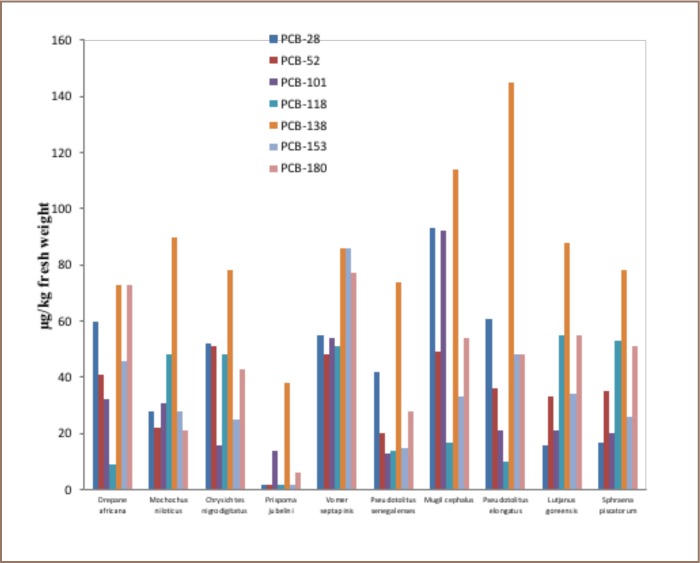
Marker PCBs in Brackish Water Fish

The concentrations of marker PCBs are presented in Figure 1. PCB-28, 52, 138, 180, 101, 118 and 153 were detected in all of the analyzed brackish water fish samples.

### Marker PCBs in Brackish Water Fish

The distribution pattern of the marker PCBs varied greatly between fish species. Marker PCB congeners PCB-138, PCB-180 and PCB-153 were most prominent in the fish samples compared to the lower chlorinated forms. PCB-28 was highly present in three fish samples: Drepane africana, Mugil cephalus and Pseudotolithus elongatus. The highest marker PCB-138 (concentration of 144.8±24.9 μg/kg) was detected in Pseudotolithus elongatus, while the lowest concentration (37.6±12.1 μg/kg) was detected Pristipoma jubelini.

The highest concentration of PCB-180 (73.2±41.7 μg/kg) was observed for Drepane africana, and the lowest concentration of 6.20±1.34 μg/kg μg/kg for Pristipoma jubelini. The highest concentration of PCB-153 (85.7±42.3 μg/kg) was detected in Vomer septapinis, and the lowest (1.71±0.98 μg/kg) in Pristipoma jubelini. For PCB-28, the highest concentration of 93.4±58.9 μg/kg occurred in Mugil cephalus, and the lowest (2.35±1.22 μg/kg) in Pristipoma jubelini. High levels of PCB-101 were also detected in Mugil cephalus, with a concentration of 92.7±108.0 μg/kg. The lighter congener PCB-52 and heavier congener PCB-118 were detected at relatively low concentrations, with a maximum concentration of 50.9±40.7 μg/kg for Chrysichthys nigrodigitatus and 55.3±49.8 μg/kg for Lutjanus goreensis.

### Co-PCBs in Brackish Water Fish

The highest mean concentrations of PCB-105 (230.0±178.1 μg/kg) and PCB-77 (85.3±28.8 μg/kg), PCB-81 (157±86.4 μg/kg) and PCB-114 (109.0±68.5 μg/kg), PCB-123 (157.0±23.5 μg/kg), PCB-118 (89.5±31.8 μg/kg) were observed in Vomer septapinis, Pseudotolithus elongatus, Pristipoma jubelini, and Sphyraena piscatorum, respectively. The lowest concentrations of PCB-105 (21.3±11.3 μg/kg), PCB-81 (25.3±4.05 μg/kg), PCB-114 (13.5±1.86 μg/kg), PCB-123 (7.22±8.72 μg/kg), PCB-77 (12.9±7.88 μg/kg), and PCB-118 (1.68±1.44 μg/kg) were largely detected in Pristipoma jubelini *(Table 2)*.

**Table 2:** Co-planar PCB Concentrations (μg/kg fresh weight) in Brackish Water Fish Samples

**Species**	**PCB-77**	**PCB-81**	**PCB-105**	**PCB-114**	**PCB-114**	**PCB-123**
Drepane africana	58.3±35.2 (23.0–93.4)	38.9±14.2 (24.7–53.1)	76.1±36.9 (39.1–113.5)	63.0±8.61 (54.4–71.6)	8.60±2.05 (6.55–10.6)	15.0±10.1 (4.78–25.3)
Mochokus niloticus	23.5±12.7 (9.66–48.9)	25.3±4.05 (18.6–30.9)	145±119.2 (41.5–385.6)	70.1±37.4 (BDL-127.0)	47.5±38.9 (4.84–117.5)	7.22±8.72 (BDL-24.7)
Chrysichthys nigrodigitatus	63.1±34.6 (21.4–115.4)	113±38.4 (55.4–159.2)	21.3±11.3 (4.28–30.2)	87.1±31.9 (50.7–135.0)	47.9±48.0 (11.5–120.3)	59.9±48.0 (11.5–12.4)
Pristipoma jubelini	12.9±7.88 (5.28–21.3)	58.0±13.5 (28.5–72.2)	37.3±12.5 (22.5–51.2)	28.3±10.3 (16.6–40.6)	1.68±1.44 (BDL-4.50)	157±23.5 (120–166.5)
Vomer septapinis	85.3±28.8 (27.5–125.0)	157±86.4 (69.3–302.5)	230.0±178.1 (48.4–493.8)	74.8±54.6 (BDL-184.5)	50.8±47.6 (7.09–146.5)	135±130.5 (24.7–396.5)
Pseudotolithus senegalensis	30.6±12.8 (17.9–43.4)	84.3±23.7 (60.0–108.0)	47.2±7.98 (39.2–55.2)	13.5±1.86 (11.7–15.4)	14.04±1.02 (13.02–15.06)	17.1±3.77 (13.4–20.9)
Mugil cephalus	51.5±25.4 (15.8–99.2)	108±41.1 (49.1–178.0)	138±151.3 (10.1–517.3)	81.6±48.5 (BDL-193.0)	16.5±8.83 (8.02–38.6)	63.2±35.5 (30.9–152.0)
Pseudotolithus elongatus	68.2±44.5 (3.77–130.0)	133±166.0 (15.5–406.4)	144±123.7 (19.02–537.8)	109±68.5 (BDL-281.3)	9.93±7.32 (BDL-25.7)	97.1±78.5 (31.5–275.0)
Lutjanus goreensis	63.5±23.8 (45.6–78.3)	75.6±7.89 (70.5–82.36)	111±88.5 (33.6–170.8)	78.5±13.6 (64.8–81.5)	88.6±21.8 (72.9–96.5)	77.5±5.61 (45.8–121.8)
Sphyraena piscatorum	60.5±21.3 (41.2–72.8)	68.6±8.69 (62.7–71.3)	102±77.5 (29.5–159)	76.8±11.5 (65.0–79.4)	89.5±31.8 (66.4–98.6)	68.6±7.89 (62.9–71.3)

The higher chlorinated dioxin like (DL)-PCBs from PCB-126 to PCB-189 occurred at relatively lower concentrations than the lower chlorinated homologues in the fish samples. The highest concentration of PCB-167 (142±45.1 μg/kg) was observed for Pristipoma jubelini and the lowest concentration of PCB-169 (2.56±1.21 μg/kg) was observed for Pristipoma jubelini *(Table 3).*

**Table 3:** Heaviest Co-PCB concentrations (μg/kg fresh weight) in brackish water fish

**Species**	**PCB-126**	**PCB-156**	**PCB-157**	**PCB-167**	**PCB-169**	**PCB-189**
Drepane africana	15.9±8.75 (7.12–24.6)	12.4±4.98 (7.42–17.4)	27.8±16.5 (11.4–44.3)	32.5±4.95 (27.6–37.5)	41.6±30.4 (11.2–71.9)	7.00±0.42 (6.58–7.43)
Mochokus niloticus	16.4±7.82 (BDL-37.7)	16.7±12.8 (BDL-42.1)	20.8±17.8 (5.36–56.5)	18.4±10.6 (BDL-32.8)	67.5±58.0 (7.30–156.0)	24.7±6.24 (1.20–72.8)
Chrysichthys nigrodigitatus	6.05±5.94 (BDL-14.9)	26.8±19.5 (5.33–56.1)	4.40±1.61 (1.98–6.38)	20.3±5.7 (11.8–28.8)	31.8±18.4 (7.97–59.4)	16.9±7.30 (9.67–27.86)
Pristipoma jubelini	25.6±12.2 (18.6–39.6)	11.8±1.56 (7.88–13.6)	3.72±0.23 (2.66–7.82)	142±45.1 (122–177)	2.56±1.21 (BDL-6.88)	3.78±1.25 (BDL-8.22)
Vomer septapinis	7.72±4.09 (BDL-12.8)	33.2±11.8 (17.9–54.8)	17.7±3.20 (14.8–24.1)	34.9±23.8 (11.6–82.4)	44.2±15.2 (18.9–61.7)	7.92±6.39 (BDL-15.3)
Pseudotolithus senegalensis	7.97±3.92 (3.25–11.1)	23.7±9.56 (14.1–33.2)	6.69±2.29 (4.40–8.98)	10.84±2.80 (8.82–13.8)	96.0±69.0 (27.0–165.0)	9.23±0.25 (8.98–9.49)
Mugil cephalus	17.7±8.71 (6.89–34.17)	83.3±26.8 (27.3–142.0)	21.7±5.07 (11.9–29.8)	37.8±5.69 (25.5–51.0)	39.0±16.6 (13.9–66.2)	11.8±2.48 (8.17–17.0)
Pseudotolithus elongatus	23.3±12.5 (BDL-36.7)	28.7±20.4 (BDL-80.2)	18.5±3.26 (13.3–23.2)	24.2±16.5 (7.36–66.4)	30.9±7.66 (21.2–41.2)	16.2±18.0 (BDL-63.5)
Lutjanus goreensis	20.8±4.56 (17.5–24.5)	24.3±3.21 (19.5–27.2)	19.4±5.55 (13.5–24.3)	32.5±7.88 (25.6–37.9)	36.7±12.2 (23.8–49.3)	13.5±4.20 (8.55–19.6)
Sphyraena piscatorum	18.6±7.54 (12.9–22.5)	23.9±4.51 (17.5–26.5)	21.8±6.43 (13.7–25.9)	30.4±9.21 (22.5–35.6)	34.8±10.3 (23.2–44.8)	15.4±11.0 (4.89–28.9)

### Total PCBs in Brackish Water Fish

The concentration sequence in the fish samples was Σ National Oceanic and Atmospheric Administration Agency (NOAA) PCBs>ΣDL PCBs>Σ International Council for the Exploration of the Seas-7 Indicator PCBs (ICES-7) PCBs. The ΣNOAA PCBs occurred at the highest concentration of 1830.0±484.0 μg/kg (range 859.0–2386 μg/kg) in Vomer septapinis, and the lowest in Pseudotolithus senegalensis, with a mean concentration of 795±169.3 μg/kg (range 626.3–964.2 μg/kg). The levels of DL PCBs were the highest in Pristipoma jubelini (992.0±88.6 μg/kg; range 788.1–1001 μg/kg), and lowest (286±81.5 μg/kg; range 204–367 μg/kg) in Drepane africana. The highest mean concentrations (418±177.6 μg/kg; range 247.4–773.2 μg/kg) of ICES-7 PCBs were observed in Vomer septapinis. The heavier ICES-7 congeners, PCB-138, PCB-153, and PCB-180 occurred at higher concentrations in all the brackish fish samples compared to the lighter molecular weight ICES-7: PCB-28, PCB-52, and PCB-101, except in Drepane africana. The European Union (EU) marker PCB limit of 335 μg/kg was exceeded in all the brackish water fishes with the exception of Mochokus niloticus, Pristipoma jubelini and Pseudotolithus senegalensis. The highest ΣPCBs 138, 153, 180 concentrations (211±40.5 μg/kg; range 174–306 < μg/kg) were detected in Pseudotolithus elongatus, and lowest concentration (45.5±24.2 μg/kg; range 34.2–66.8 μg/kg) in Pristipoma jubelini.

The highest sum of low molecular weight ICES-7 PCBs: 28, 52, and 101 was detected in Mugil cephalus (187±86.4 μg/kg; range 47.4–345.5 μg/kg), and the lowest in Pristipoma jubelini (15.8±12.5 μg/kg; range 5.34–30.6 μg/kg). The total level of PCBs in the brackish fish samples was relatively high, at >1000 μg/kg (The World Health Organization (WHO) and Food and Agriculture Organization of the United Nations (FAO) (2001) guideline is 1000 μg/kg fresh weight). The sum PCB concentration sequence in the brackish fish samples was Vomer septapinis > Lutjanus goreensis > Sphyraena piscatorum > Pseudotolithus elongatus > Mugil cephalus > Chrysichthys nigrodigitatus > Mochokus niloticus > Pristipoma jubelini > Drepane Africana > Pseudotolithus senegalensis. In addition, the fish species accumulated more of the heavy marker PCBs compared to the lighter ones. The EU guideline value for fish (220 μg/kg fresh weight) was exceeded in about 80% of the studied fish samples.

The 12 dioxin-like (DL)-PCBs were analyzed in the brackish water fish samples during the study. The congeners were PCB-77, PCB-81, PCB-105, PCB-114, PCB-118, PCB-123, PCB-126, PCB-156, PCB-157, PCB-167, PCB-169, and PCB-189.

Table 6 shows the coplanar PCBs that have been assigned toxicity equivalent factors (TEFs) by the WHO.

Compared to PCBs-77, 81, 105, 114, 118 and 123 congeners *(Table 2)*, the concentrations of congeners PCB-126, PCB-156, PCBs-157, PCBs-167, PCBs-169 and PCB-189 levels were low *(Table 3).* The sequence of the highest PCB concentration levels in brackish water fish were PCB-105> PCB-123> PCB-81> PCB-114> PCB-118> PCB-77 (AS 230.0±178.1> 157±23.5> 157±86.4> 109±68.5> 89.5±31.8> 85.3±28.8).

Three PCB types studied in brackish fish species were National Oceanic and Atmospheric Administration Agency (NOAA) PCBs, DL PCBs or co-planar PCBs and marker or International Council for the Exploration of the Seas-7 (ICES-7) PCBs *(Tables 4 and 5).*

**Table 4:** ΣMarker, ΣCo-Planar and ΣNOAA PCBs (μg/kg fresh weight)

**Species**	**ΣPCB, 28, 52, 101**	**ΣPCB, 138,153, 180**	**ΣCo-PCB**	**ΣMarker PCB**	**ΣNOAA PCB**	**ΣPCBs**
Drepane africana	133±39.5 (93.9–173.0)	127±49.3 (78.4–177.0)	286±81.5 (204–367)	269±311.2 (179.0–361.4)	805±91.0 (585.1–1026)	1080±123.4 (764.3–1387)
Mochokus niloticus	61.9±24.8 (36.3–101.2)	122±31.5 (75.1–184.0)	519±101.1 (317–613)	258±40.6 (204.5–339.8)	974±304.1 (599.2–1357)	1230±318.6 (856.3–1561)
Chrysichthys nigrodigitatus	119±93.6 (39.3–259.0)	134±31.6 (85.8–171.0)	552±80.8 (474.3–673.8)	319±64.7 (240.1–415.7)	1130±160.4 (953.0–1373)	1430±159.6 (1190–1619)
Pristipoma jubelini	15.8±12.5 (5.34–30.6)	45.5±24.2 (34.2–66.8)	992±88.6 (788.1–1001)	61.2±32.6 (58.9–99.6)	1080±90.5 (928.0–1206)	1140±125.3 (850.6–1244)
Vomer septapinis	156±49.8 (57.2–252.6)	209.1±82.6 (133–376)	937±456.3 (465–1448)	418±177.6 (247.4–773.2)	1830±484.0 (859.0–2386)	2240±540.3 (1163–2748)
Pseudotolithus senegalensis	74.6±12.0 (62.6–86.7)	125±6.50 (118–131)	358±34.5 (323.5–392.4)	213±19.5 (194.1–233.4)	795±169.3 (626.3–964.2)	1010±188.3 (820.7–1197)
Mugil cephalus	187±86.4 (47.4–345.5)	199±47.8 (80.4–276.5)	768±321.5 (352.4–1533)	403±132.8 (200.4–587.3)	1280±400 (632.7–2199)	1680±514.0 (907.6–2785)
Pseudotolithus elongatus	117±46.3 (40.8–164.5)	221±40.5 (174–306)	720±200.5 (381.0–1464)	348±63.4 (259.2–489.1)	1640±420.2 (1040–2105)	1980±400.5 (1388–2594)
Lutjanus goreensis	121±55.6 (86.5–177.6)	189±44.56 (143–197)	724±102.3 (657–823.8)	403±32.9 (378.2–440.8)	1720±234.0 (1600–2001)	2170±560.8 (1967–2456)
Sphyraena piscatorum	115±43.2 (65.9–168.5)	178.6±24.5 (147–188)	691±22.6 (640.2–712.7)	381±45.0 (320.1–418.6)	1820±201.1 (1600–1835)	2000±230.1 (1789–2231)

**Table 5:** Description of PCB types

**PCB Types**	**Description**
**NOAA Co-planar PCBs**	NOAA (National Oceanic and Atmospheric Administration) PCBs are namely DL (Dioxin-like) PCBs or Co-planar PCBs and Marker PCBs. The NOAA PCBs include 18 congeners; PCB-8, PCB-18, PCB-28, PCB-52, PCB-44, PCB-66, PCB-101, PCB-105, PCB-118, PCB-128, PCB-138, PCB-153, PCB-170, PCB-180, PCB-187, PCB-195, PCB-206 and PCB-209. The NOAA PCBs were recommended for monitoring as indicators due to their relatively high concentration in technical mixtures and their wide chlorination range (2–10 chlorine atoms per molecule).
**NOAA PCBs**	The co-planar or dioxin-like PCBs include 3 non-ortho (PCB 77, PCB 126 and PCB 169), 8 mono-ortho (PCB 105, PCB 114, PCB 118, PCB 123, PCB 156, PCB 157, PCB 167 and PCB 189) and 2 *diortho* (PCB 170 and PCB 180) substituted congeners which have been shown in experimental systems to exert a number of responses similar to those observed for 2, 3, 7, 8-TCDD^28^
**Marker PCBs**	Marker PCBs, sometimes called “Dutch seven” or ICES7, are seven PCBs that have been measured for many years as an indication of the total PCB contamination. They include PCB-28, PCB-52, PCB-101, PCB-118, PCB-138, PCB-153, and PCB-180. One of these seven, PCB118, is classified as a dioxin-like PCB

**Table 6:** Brackish Water Fish Toxic Equivalent ng/kg and Acceptable Daily Intake

**Species**	TEQ^*^	ADI^*^	**ΣPCBs**	**% Fat**
Drepane africana	72.6±6.44	1.21	1080±123.4	1.22±0.94
Mochokus niloticus	68.3±8.20	1.13	1230±318.6	4.04±1.08
Chrysichthys nigrodigitatus	91.6±9.56	1.52	1430±159.6	6.12±0.78
Pristipoma jubelini	36.3±20.5	0.61	1140±125.3	1.18±0.19
Vomer septapinis	129±13.5	2.15	2240±540.3	3.42±2.16
Pseudotolithus senegalensis	42.3±21.2	0.71	1010±188.3	4.62±0.22
Mugil cephalus	121±3.26	2.00	1680±514.0	3.24±1.38
Pseudotolithus elongatus	126±8.35	2.08	1980±400.5	2.32±1.46
Lutjanus goreensis	105±8.56	1.74	2170±560.8	6.60±1.12
Sphyraena piscatorum	157±13.5	2.60	2000±230.1	2.25±1.33

**Abbreviations:** ADI, acceptable daily intake; TEQ, toxic equivalent

^*^TEQ in ng (TEQ) kg^−1^, ADI in ng (TEQ) kg^−1^/body weight (bw)/day.

## Discussion

Non-ortho DL PCBs 77, 81, 126, and 169 have been extensively discussed in the literature. Most of the levels found in fresh fish are very low compared to the findings in the present study. New Zealand eel and trout fillets contained very low levels of PCB-77, with ranges of <0.001 to <0.008 μg/kg fresh weight.^13^,^14^ Paasivirta *et al.* detected slightly elevated levels in Finland salmon, at 1.50 to 18.50 μg/kg.^15^ Williams detected low levels (0.46 to 5.34 μg/kg) in Chinook salmon from Lake Michigan.^16^ The levels detected in the present study (25.5 to 125.0 μg/kg fresh weight) for Vomer septapinis were high compared to global ranges.

In addition, very low levels of PCB-126 (range <0.001 to 1.97 μg/kg fresh weight) were detected in trout and eel fillets from New Zealand, rainbow trout and salmon from Finland, and char white fish from Sweden.^13–15^ The levels detected in the present study (18.6 to 39.6 μg/kg fresh weight) in Pristipoma jubelini are high compared to global ranges.

Very low levels of PCB-169 were detected in Chinook salmon from Lake Michigan, USA (<0.008), eel from various rivers in the Netherlands (0.003 to 0.24 μg/kg fresh weight), and <0.1 to 0.63 μg/kg fresh weight in salmon from Finland.^15–17^ The levels of PCB-169 detected in the present study (27.0 to 165.5 μg/kg fresh weight) in Pseudotolithus senegalensis were high compared to global ranges. This has very serious health implications for fish consumers.

High levels of penta- and hexachlorobiphenyls DL PCBs PCB-105, PCB-118 and PCB-156 were detected in fishes globally. PCB-105 at a concentration of 0.35 to 170 μg/kg was detected in eel from Finland, 1.90 to 110 μg/kg in eel from a clean lake in the Netherlands, and 110–100 μg/kg in salmonids from Lake Ontario.^15^,^17^,^18^ In addition, high levels of PCB-118 have been reported in the literature. Paasivirta *et al*. detected high levels of PCB-118 in salmon from Finland (190 to 340 μg/kg fresh weight) and 7.90 to 340 μg/kg fresh weight was observed by de Boar *et al*. in eel from lakes in the Netherlands.^15^,^17^ In the present study, lower levels (66.4 to 98.6 μg/kg fresh weight) were detected in Sphyraena piscatorum. PCB-138 was present at a high concentration, consistent with data from other countries. The most prevalent mono ortho-PCBs in this study were PCB-81, PCB-105, PCB-114 and PCB-118, similar to results in other countries.

The available literature reported that total PCB concentrations in fish from Europe and North America exceed 100 μg/kg, in agreement with the maximum PCB concentration reported in the present study. One of the most studied contaminated freshwater ecosystems in North America is the Great Lake ecosystem. The Great Lake ecosystem, like the River Niger, receives a wide variety of pollution discharge from the numerous industrial, agricultural and population centers on its shores. High levels of PCB contamination in the Great Lakes have been reported for trout and salmon muscle, reaching 4300–10,000 μg/kg.^19^ However, brown trout from Michigan had PCB concentrations of only 20–6,000 μg/kg.^16^ These results were similar to the levels observed in this study: Vomer septapinis, 1163–2748 μg/kg; Mugil cephalus, 907.6–2785 μg/kg; Lutjanus goreensis, 1967–2456 μg/kg; and Sphyraena piscatorum, 1789–2231 μg/kg.

Most regulatory criteria for PCBs in fish are aimed at the protection of human health. In North America, including Canada, fish containing levels of PCBs greater than 2000 μg/kg are considered unsuitable for human consumption. Other limits are more stringent, such as the FAO, WHO and Switzerland at 1,000 μg/kg. All of the fish samples in this study were above these more stringent regulations. Available criteria for the protection of aquatic life based on tissue residue concentrations are lower than those for the protection of human health. Maximum tissue residue concentrations of 100, 110, and 500 μg/kg apply in British Columbia, New York and Australia, respectively. The levels quantified in the present study were 10- to 20-fold above these criteria.

Osibanjo and Bamgbose reported contamination by chlorinated hydrocarbons (CLHC) of Nigerian marine fish and shell fish, based on analyses of 94 samples of 25 marine fish species between 1983–1985.^20^ They detected low PCB levels of 0.70–14.0 μg/kg. Adeyemi *et al*. analyzed several fish samples from the Lagos lagoon in Nigeria for the presence of PCBs.^21^ The analyzed fish species included Tilapia zilli (redbelly tilapia), Ethmalosa fimbriata (bonga shad) and Chrysichthys nigrodigitatus (catfish). Eight PCB congeners were identified and quantified in muscle of the analyzed species. The concentration of total PCBs in samples ranged from 560 to 2940 μg/kg. These levels were close to the levels detected in the present study.

### Toxic Equivalent and Acceptable Daily Intake

The UK Committee on Toxicity of Chemicals in Food, Consumer Products and the Environment (COT) has recently recommended the use of toxicity equivalent factors to assess the potential toxicity of complex mixtures of dioxin and the coplanar PCBs present in food (Consumer Products and the Environment, COT 1997). An acceptable daily intake of 10 pg toxic equivalent TEQ/kg bw/day 27 can be used to assess the health risks of the intake of mixtures of polychlorinated dibenzo-p-dioxins/furans (PCDDs/F and PCB congeners. The toxicity equivalents concept uses available toxicological and biological data to generate a set of weighing factors (TEFs), each of which expresses the toxicity of ‘dioxin-like’ compounds in terms of the equivalent amount of 2, 3, 7, 8- tetrachlorodibenzo-p-dioxin (TCDD). Multiplication of the concentration of a congener by its TEF gives a TCDD Equivalent or TEQs.^22^,^23^

The PCB TEQs were derived using the 1994 WHO TEF values.^24^ It should be noted that PCB congeners, especially the mono *ortho*-PCBs induce hardly any biological or toxic response in fish, in distinct contrast with mammals. This is reflected in the new TEFs recently established by the WHO.^25^

The highest TEQ (157 ng TEQ kg^−1^) was detected in Sphreana piscatorum. Lower but moderately high TEQ values were detected in Chrysichthys nigrodigitatus, Drapane africana, Mochocus niloticus, Pseudotolitus senegalenses, and Pristipoma jubelini, with TEQ values of 91.6±9.56 ng TEQ kg^−1^, 72.6±6.44 ng TEQ kg^−1^, 68.3±8.20 ng TEQ kg^−1^, 42.3±21.2 ng TEQ kg^−1^, and 36.3±20.5 ng TEQ kg^−1^, respectively. The lower TEQ values corresponded with the following acceptable daily limits (ADI): 1.52, 1.21, 1.13, 0.71 and 0.61 ng TEQ/kg/bw/day, respectively. Dioxin-like congeners 114 and 156 are the highest contributors to the TEQ values in the brackish water fish.

An acceptable daily intake of 10 pg TEQ/kg bw/day can be used to assess the health risks of the intake of mixtures of PCDDs/Fs and PCB congeners. This ADI, which has been adopted by several countries, including Canada and the Netherlands, has been revised downwards by the WHO to a range of 1–4 pg TEQ kg bw/day. The Health Council of the Netherlands has specified a health-based exposure limit of 1 pg TEQ/kg bw/day. In addition to these ADIs, some countries have regulations for dioxin-like PCB concentrations or international TEQ in specific food types (summarized in Buckland *et al.*).^13^ In the United States, a limit value has been set for dioxins in fish. This guideline recommends not consuming fish with dioxin levels greater than 25 ng TEQ kg^−1^ on a wet weight basis (Food and Drug Administration, cited in the US Environmental Protection Agency (USEPA), 1987). In Ontario, there is a guideline value for sport fish of 15 ng TEQ kg^−1^. The regulation of dioxins and furans for the protection of wildlife is complicated by the bioaccumulative nature of these compounds. To address this issue, the Canadian Council of Ministers of the Environment has proposed both a tissue residue guideline (50 ng I-TEQ kg^−1^ fat) and a dietary intake guideline (1.1 ng I-TEQ kg^−1^ fresh weight) for the protection of aquatic wildlife.^26^ The TEQ values in brackish water fish from this study exceeded the regulatory limits in all of the fish samples and the ADI values also exceeded the regulatory limits in all of the samples, with the exception of 0.61 ng I-TEQ kg^−1^ and 0.71 ng I-TEQ kg^−1^detected in Pristipoma jubelini and Pseudotolitus senegalensis, respectively.

## Conclusions

The total PCB levels in the brackish fish samples were relatively high at >1000 μg/kg, above the WHO/FAO guideline of 1000 μg/kg fresh weight. The EU guideline value for fish of 220 μg/kg fresh weight was exceeded in about 80% of the brackish water fish samples. Consumption of fish from the River Niger may expose humans to polychlorinated biphenyls. In addition, since contamination of the fish samples was an indication of river contamination, water quality is of great concern and there is a need for additional PCB data on water quality to be distributed to the community, followed by mitigation measures.
